# The growing season of Poland in the changing climate based on phenological observations

**DOI:** 10.1007/s00484-025-03037-9

**Published:** 2025-09-25

**Authors:** Małgorzata Szwed, Joanna Chmist-Sikorska, Małgorzata Kępińska-Kasprzak

**Affiliations:** 1 Department of Meteorology and Climatology, Institute of Meteorology and Water Management—National Research Institute, Podleśna 61, 01-673 Warszawa, Poland; 2 Specialist Forecasting Department, Institute of Meteorology and Water Management—National Research Institute, Podleśna 61, 01-673 Warszawa, Poland; 3 Poznań, Poland

**Keywords:** Climate change, Phenology, Growing season shift, Very early spring, Autumn

## Abstract

The growing season is the period when weather conditions (e.g., precipitation, temperature, wind, etc.) in a given area support plant growth and development. This study examines how climate change has influenced the growing season duration in Poland over the past nearly 80 years, based solely on phenological observations. The research was conducted for two 15-year periods: the pre-warming period (1946–1960), before significant global climate warming became evident, and the warming period (2007–2021), characterized by the additional influence of the “greenhouse component” on climate trends. The former dataset was sourced from the Yearbooks of Phenological Observations, which had not been previously available to a wider audience and were digitized by the authors for this study. The latter dataset was obtained from the database of the Institute of Meteorology and Water Management–National Research Institute. The results indicate an increasingly earlier onset of the vegetation period and a slight delay in its end over time, leading to an extended growing season. Today, as a result of climate change, its duration has increased to over 240 days in the west, while in the central lowlands and the Lublin Upland, it has reached 220–230 days. However, it remains almost unchanged along the coast and in northeastern Poland. Due to its location in a transitional temperate climate zone, Poland experiences high weather variability, which is also reflected in fluctuations in the start dates and duration of growing seasons.

## Introduction

Phenological research (mainly phytophenological ones) is used in many natural sciences and has broad practical applications. It aids in studying the geographical environment, particularly in recognizing/understanding the interactions between living organisms and the environment and understanding ecological processes (Walther et al. 2002).

Studies on the relationship between the annual cycle of plant development on Earth and elements of the natural environment have shown that, despite the significant influence of factors such as soil type or biospheric factors (e.g., pests), the most critical determinants of the seasonality of plant development cycles and their spatial and temporal variations are air temperature, day length, sunshine, and precipitation/humidity level. Thus, a close relationship exists between individual phases of plant development and meteorological conditions. Plants continuously respond to all weather phenomena, not just a single variable (e.g., temperature). They provide information on ecosystem response to seasonal climatic conditions, and they respond to climate change in the long term. Consequently, phytophenological observations serve as a valuable complement to direct meteorological measurements, and their results play a crucial role in detecting climate change (Kalvãne et al. 2009; Ovaskainen et al. 2013; Menzel et al. 2020; Singh et al. 2023). Undoubtedly phenological observations and research studies are becoming increasingly important, although some scientists argue that existing data series should be treated with caution due to their relatively short duration (e.g., Cerlini et al. 2022), examples in the literature demonstrate climate change’s undeniable impact on ecosystems’ seasonality.

Changes in climatic conditions, both globally and regionally, observed since the late 20th century have led to various efforts to document their impact on the natural environment, including the plant world. In Europe, it has been noted that plants’ early spring development phases, which also mark the start of the growing season, tend to occur earlier in recent years despite a wide range of variability in dates. Simultaneously, a slight delay at the end of the growing season in some regions, combined with its earlier onset, has gradually expanded the growing season. In the Discussion section, the authors will discuss studies on shifts in and the duration of the growing season in other regions, with a particular focus on the European continent.

In the context of climate change, analyses of vegetation period parameters play an important role. The growing season is the period when most or all plants in the area under consideration undergo their annual development cycle. So, it is when weather conditions (e.g., precipitation, temperature, and winds) in a given area support plant growth and development. In Poland (generally in middle latitudes), this period is usually defined as when the average daily air temperature exceeds 5 °C (Koźmiński and Michalska 2008).

From the phenology point of view, the growing season begins in very early spring when trees and shrubs awaken from winter dormancy and develop flower buds before their leaves emerge. The end of the growing season coincides with phenologically proper autumn when plants prepare for winter rest. To encompass the vegetation periods of as many plants as possible in a given area, the start of the growing season should be marked by the earliest phenological phase in spring. At the same time, its end should correspond to the latest phase in autumn. It is generally assumed that the cut-off dates of phenological seasons are best determined based on observations of wild-growing plants, i.e. plants whose development is not influenced (or is only minimally influenced) by human activity (e.g. agrotechnical treatments). At the same time, these plants should be widespread throughout most of the observed area. The selection of indicator species for Polish conditions was proposed by Łastowski (1951).

In practice, the cut-off dates for subsequent phenological seasons are determined based on the average long-term date for several plants selected from among the guide (indicator) plants characteristic of a given phenological season. Sometimes, the dates for only one key plant are considered (if only one plant is available). While including a larger number of plants provides a broader understanding of climatic conditions through their phytophenological responses, constructing maps based on multiple plants can risk obscuring differences between different parts of the mapped area by excessively averaging the influence of climate on various species. Unfortunately, selecting indicator species does not always mean that the same plants and their appearances are considered in phenological studies. Different researchers, guided by general guidelines, individually select indicator species and their occurrences for a given area, depending on the presence of indicator species. Sometimes, the selection changes over the long term if one indicator species disappears due to various circumstances, such as changes in land use, etc.

This research aims to verify the hypothesis about extending the growing season due to climate change based on phenological data. It will describe the variability of the growing season, one of the climate change symptoms, in Poland over almost 80 years. This study will analyze phytophenological occurrences marking the start and end of the growing season during two 15-year periods, historical 1946–1960 and present 2007–2021, at 35 observation stations in Poland. Historical data stem from phenological annuals, The Yearbooks of Phenological Observations from 1946 to 1960, accessed by the authors, contain highly valuable observational data, enriching the understanding of climatic conditions before the onset of the greenhouse signal. This material has so far been used mainly internally by the Institute, and its results have not been published/made available to a broader group of researchers. While these two 15-year-long data series (due to their length) provide only a preliminary and approximate assessment of changes in the timing of individual phenological phases (and/or seasons), their comparison can offer a clearer understanding of the climate changes that have occurred over the past 80 years. This research will enhance knowledge of Poland’s climate change, including its trends/tendencies and spatial variability. At the same time, this research helps fill Poland’s blank spot on the map of European phenology.

## Data and methods

**Fig. 1:**
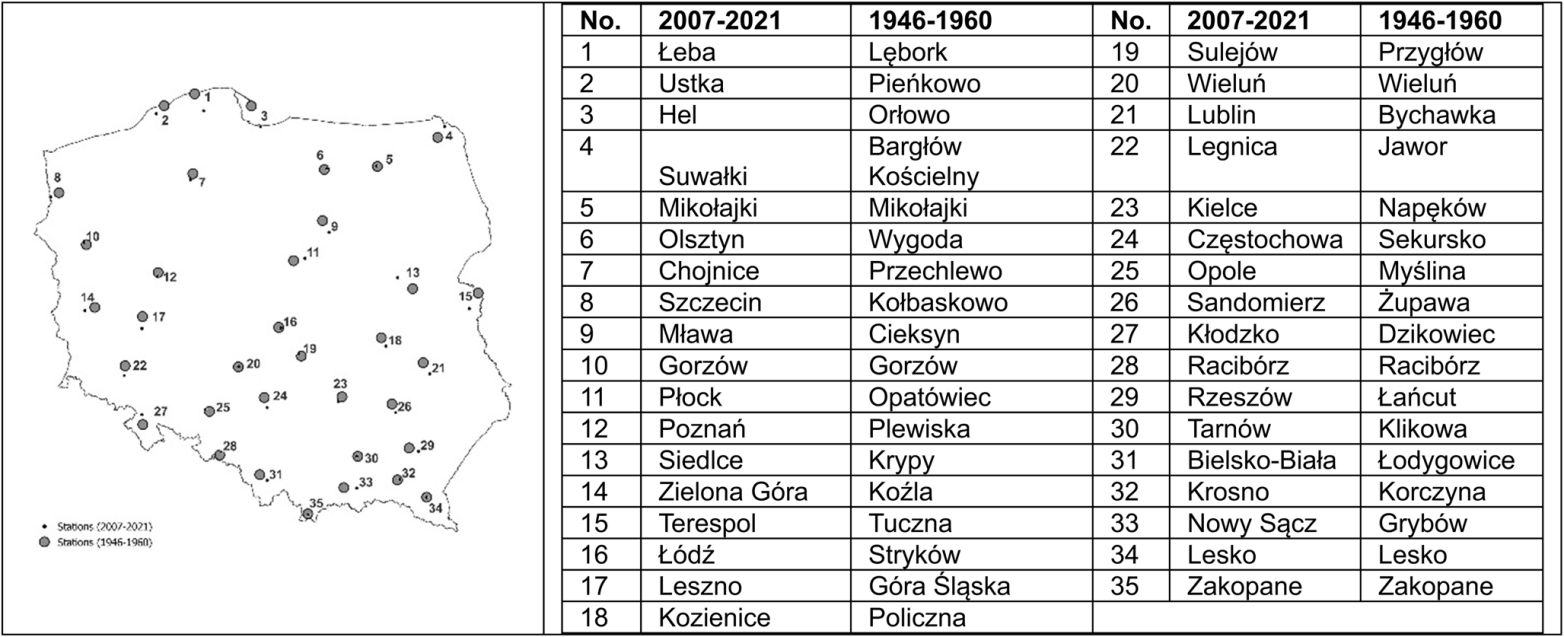
Phenological observation posts 2007–2021 and their counterparts from 1946 to 1960

The article examines the variability of the growing season over the past nearly 80 years, based on phenological events recorded during two 15-year observation periods, 1946–1960 and 2007–2021, at 35 observation stations. Figure 1 shows the location of 35 observation posts selected for analysis, which are part of the current network of phenological observations, along with their counterparts/matches from 1946 to 1960. Daily average air temperature data from 1951 to 1960 for 35 meteorological stations were used to verify the phenological database available in the printed yearbooks, which were also sourced from the public database of the Institute.

For this analysis, the start dates of two phenological seasons, i.e., very early spring and proper autumn—marking the boundaries of the growing season—were determined. Specifically, the research utilized annual datasets of 4 phenological events from 1946 to 1960 and 2007–2021. Phenological events used for analysis include hazel flowering (*Corylus avellana L.*), coltsfoot flowering (*Tussilago farfara L.*), birch leaf color change, and birch leaf falling (*Betula pendula Roth*).

The hazel and coltsfoot flowering were used to define the start of very early spring (which also marks the first day of the vegetation period). In contrast, the color change of the birch leaf and its falling were used to define the start of proper autumn (which marks the last day of the vegetation). The start date of the phenological season is the arithmetic mean of the dates of specific occurrences.
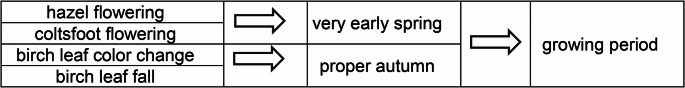


The dates of individual phenological events, the beginning and the end of the growing season, and its duration in each subsequent year, as well as across the analyzed 15-year periods, were studied. Both 15-year periods were compared. The variability of the growing season’s beginning and end was discussed under the so-called normal conditions for every 15 years and in years with extreme phenological conditions.

To ensure the reliability and consistency of the analyzed time series 1946–1960, the Standard Normal Homogeneity Test (SNHT) was applied to detect potential inhomogeneities in the data. The test compares the candidate station’s data with reference series constructed from neighboring stations, using normalized differences to detect deviations. The SNHT computes the T-statistic for each potential breakpoint in the series, and values ​​exceeding the critical threshold indicate significant inhomogeneities. In this study, due to the scale of the data, the threshold was adjusted to account for more minor variations in the time series. T values below 0.6 indicated homogeneous data, while higher values ​​suggested potential breakpoints. At the same time, data quality was verified by comparing the dates of specific occurrences with the course of average air temperature at the nearest synoptic station. Phenological stations that failed the test and/or those with obviously erroneous observations (e.g., when a phenological event indicating the beginning of vegetation was recorded while the average air temperature for the preceding 20 days was below zero) were excluded. After the selection process, 35 observation stations from 2007 to 2021 and their counterparts from 1946 to 1960 were selected for analysis.

QGIS software version 3.20, with the SAGA plugin enabling value interpolation using the spline method, was used to illustrate the spatial distribution of the occurrence dates of individual phenological events and/or phenological seasons. The input data was processed and optimized for spatial analysis to represent the spatial variability of the studied parameters accurately. However, given that hypsometry and topography play a dominant role in the course of meteorological indicators in mountainous areas, extrapolation values in these regions should be treated with great caution or even considered as potentially erroneous.

To evaluate whether the differences in the timing of the start and end of the growing season between the historical period (1946–1960) and the present period (2007–2021) were statistically significant, Welch’s t-test was applied for each location with available data from both periods. The test was selected for its robustness to unequal variances and sample sizes. Mean differences and p-values were calculated separately for the onset and end of the growing season.

### Approach to the selection of time series

Phenological observations have been conducted regularly at special network of the Institute of Meteorology and Water Management (formerly National Hydrology-Meteorology Institute) since 1946. However, the number of observation stations gradually decreased over time, and in 1992, the observations were permanently suspended. From this period, only data from the years 1946–1960 have been preserved, published in Yearbooks of Phenological Observations. In 2007, the Institute resumed phenological monitoring, which has since been carried out systematically in the vicinity of the IMGW-PIB synoptic stations. As a result, this study was able to analyze two available 15-year observation periods: 1946–1960 and 2007–2021.

### Approach to the selection of observation stations

Phenological observations in the 1940 s and 1950 s were usually carried out in different locations than those in recent years. The study aims to analyze ongoing climate changes from a broader perspective rather than focusing on changes in specific locations. To achieve this, older observation sites were matched to current stations. The base locations were the stations where observations are currently conducted. To ensure the best possible matching, an area with a 30 km diameter (buffer) was generated around each current station to locate the nearest historical stations within this range. If multiple stations were found within a given buffer (often the case), the selection was further refined based on the quantity of data available. Only stations that recorded a specific phenological occurrence in at least 10 of the 15 observed years were included. Each occurrence was analyzed separately since not all stations, past or present, conducted (and currently conduct) the full range of observations.

The number of analyzed stations for the specific occurrences was as follows: hazel flowering – 35, coltsfoot flowering – 35, birch leaf color change – 35, and birch leaf fall – 31 stations. The given numbers, e.g., hazel flowering – 35, indicate the maximum number of stations available for analysis for a given occurrence. In some cases, not all stations (the current station and its pre-1960 counterpart) have sufficient/homogeneous data in both analyzed fifteen-year periods. As a result, the number and set of stations examined may vary in each case. The number of analyzed stations was smaller for determining the dates marking the beginning of very early spring and proper autumn, as only stations with data on two relevant events could be used. Thus, the date marking the start of vegetation, based on the combined dates of hazel and coltsfoot flowering, was analyzed at 26 stations. The end of vegetation, determined using two birch-related events, was analyzed also at 26 stations (but not the same as in the case of the vegetation start). Four phenological events (two at the beginning and two at the end of the growing season) in a specific location were required to calculate the duration of the growing season. Consequently, the number of analyzed stations containing all the necessary observations was reduced to 19.

The authors deliberately chose not to restrict all analyses to these 19 stations to enhance spatial analyses. At each analysis stage, they ensured that additional stations did not distort the average values for Poland compared to analyses limited to 19 stations. It is important to note that rigidly adhering to the assumption that only stations with all four required events in complete sequences should be analyzed would have made the research impossible. On the other hand, processing the original data by filling in gaps using statistical methods — such as those applied in some meteorological time series (e.g., for average temperature) — would have compromised the original and unique character of data derived from phytophenological events.

### Approach to the selection of indicator plants and plant events

For the authors of this paper, the starting point was the methodology and, thus, the selection of indicator plants and the occurrences currently observed in the Institute’s network. The beginning of the very early spring, and therefore the growing season, is determined based on the blooming of female flowers of common hazel (*Corylus avellana L.*) and common coltsfoot (*Tussilago farfara L.*). This developmental phase of both species was also observed during the historical period. In contrast, autumn onset (i.e. the end of the growing season) is currently determined at the Institute based on the yellowing and falling leaves of small-leaved lime (*Tilia cordata Mill.*) and silver birch (*Betula pendula Roth*). Until recently, autumn was also determined by another phenomenon, namely the yellowing of the leaves of the horse-chestnut tree (*Aesculus hippocastanum L.*). In recent years, this practice had to be abandoned due to the invasion of the horse-chestnut leaf miner (Cameraria ohridella), a moth pest of horse-chestnut trees. Without entering into a debate on the damage caused by the leaf miner or whether its invasion leads to tree mortality, it can be stated with certainty that the leaf miner causes the late-summer browning of leaves, which cannot be confused with the arrival of autumn. From 1946 to 1960, no data were collected on the yellowing and falling of lime leaves.

For the reasons mentioned above, this paper determines the beginning of the very early spring by the methodology developed over the years at the Institute, based on the flowering of hazel and coltsfoot. In contrast, autumn is determined solely based on two phenomena observed in birch.

## Results

### Beginning of vegetation

On average, in Poland, the beginning of very early spring during the multi-year period 1946–1960 —determined based on 26 phenological stations—occurred on the 84th day of the year (March 23), while after 2007, it occurred eleven days earlier, i.e., on the 73rd day of the year (March 12). These are the average values for Poland (in the analyzed 15-year periods) and vary depending on the location. The onset of vegetation accelerated at 25 out of 26 studied stations. The acceleration of the growing season was not observed only on the coast.

**Fig. 2:**
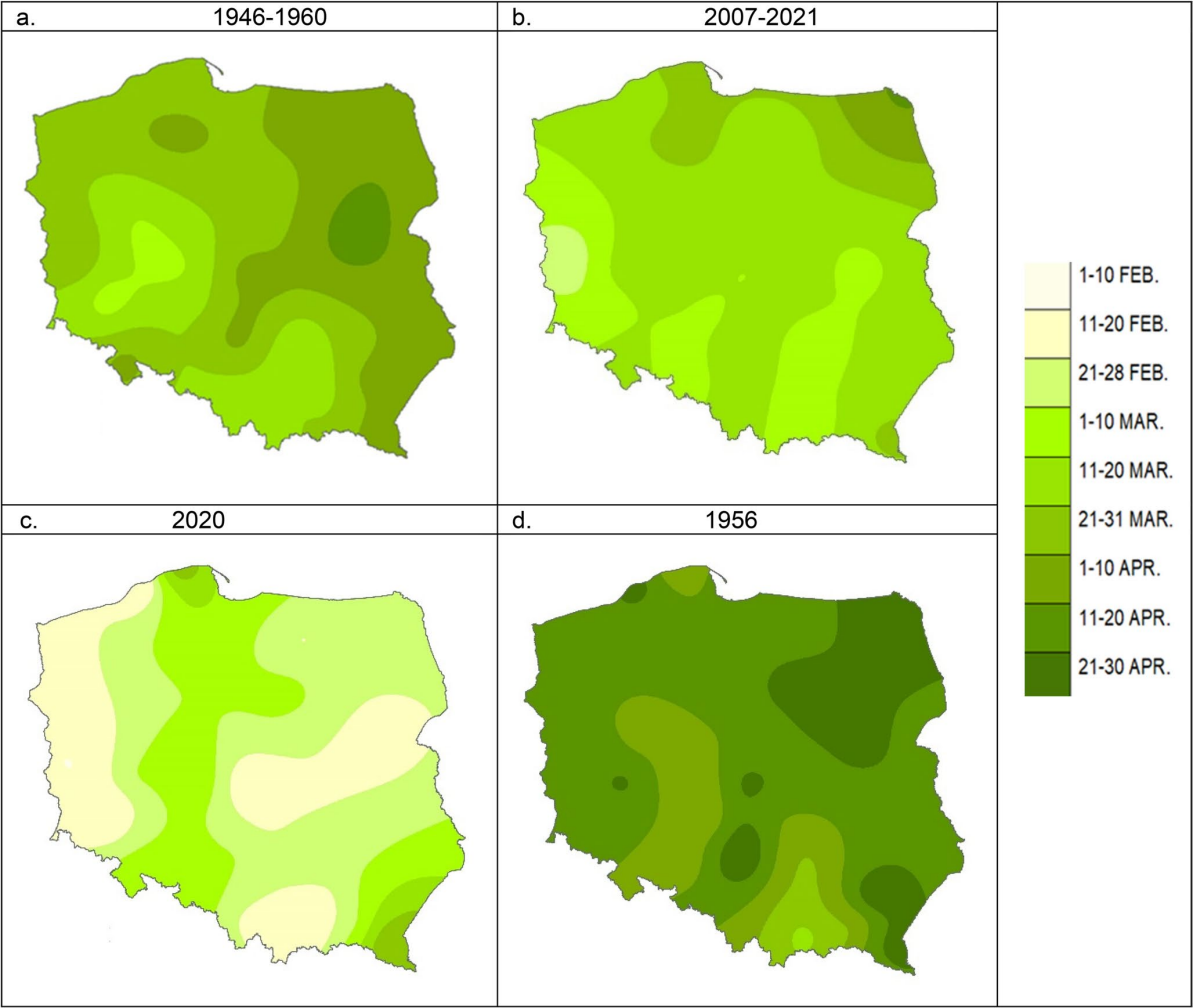
The beginning of the very early spring in 1946–1960 (**a**), 2007–2021 (**b**), 2020 (**c**), and 1956 (**d**) as [date]; Source: own study

Phenologically, very early spring enters Poland from the southwest, gradually advancing towards the country’s northeast. Before 1960, this progression began at the end of the first decade of March, covered half of the country by early April, and reached the furthest borders in the second half of April. After 2007, vegetation begins in the west and southwest as early as the end of February. It advances into the interior of the country at a faster pace, reaching most of the territory by the end of March (Fig. 2a and b).

The factors disturbing the average onset and progression of very early spring for selected species include a long and frosty winter with thin snow cover, low air temperatures and precipitation in spring (delayed vegetation), as well as high winter and spring temperatures, preferably accompanied by high air and soil humidity (accelerated vegetation). Figure 2c and d show the isophenes of the onset of very early spring for the year 2020 when it came exceptionally early, and the year 1956, when vegetation started exceptionally late. High temperatures in the preceding months primarily caused the exceptionally early onset of very early spring in 2020. Average temperatures in the winter of 2019/2020 (i.e., December-January-February) were significantly above normal across Poland. The average temperature deviation from the norm (1971–2000) exceeded 2.0 °C, with the highest recorded in the Suwałki region and/or the Lublin region (over 4 degrees). As a result, snow cover persisted only in the high mountain areas, while in the lowlands and uplands, it appeared only in trace amounts for several to a dozen or so days throughout the entire winter. March was also warmer than usual across Poland. Different humidity conditions ultimately influenced the spatial variability in the onset of very early spring.

In 1956, the onset of spring came exceptionally late. Below-normal temperatures marked the preceding winter season, although they were not record lows, even when considering only the 30 years of this study. During the analyzed period, the coldest was the winter of 2009/2010 in most of the country. However, the transition from winter to spring was significantly colder than usual. In addition, the snow cover lasted an extended period and was among the thickest recorded in the years analyzed. It persisted particularly long in the northeastern parts of Poland.

The results of the t-Welch’s t-test showed that, among the 26 analyzed stations, eight locations exhibited statistically significant differences in the start date of the growing season (*p* < 0.05), with the most pronounced change occurring in Zielona Góra, where the season now begins, on average, a month earlier (*p* < 0.0001). Other stations with statistically significant shifts in the growing season are located in western, southwestern, and southern Poland. Across most of the country, however, the differences in the timing of vegetation onset are weaker and not statistically significant, indicating only certain tendencies toward an earlier start of the growing season.

### End of vegetation

On average, proper autumn in Poland — determined based on 26 phenological stations — occurred on the 288th day of the year (September 15) during the period 1946–1960. After 2007, this date shifted by only four days (to September 19), primarily due to the later falling of birch leaves, while the yellowing date remained almost the same. A delay in proper autumn was recorded at 19 out of 26 analyzed stations. Stations where no changes or even a slight acceleration in the arrival of autumn were observed are located in the north and in the southwestern part of the country (in the Klodzko Valley and surroundings).

**Fig. 3:**
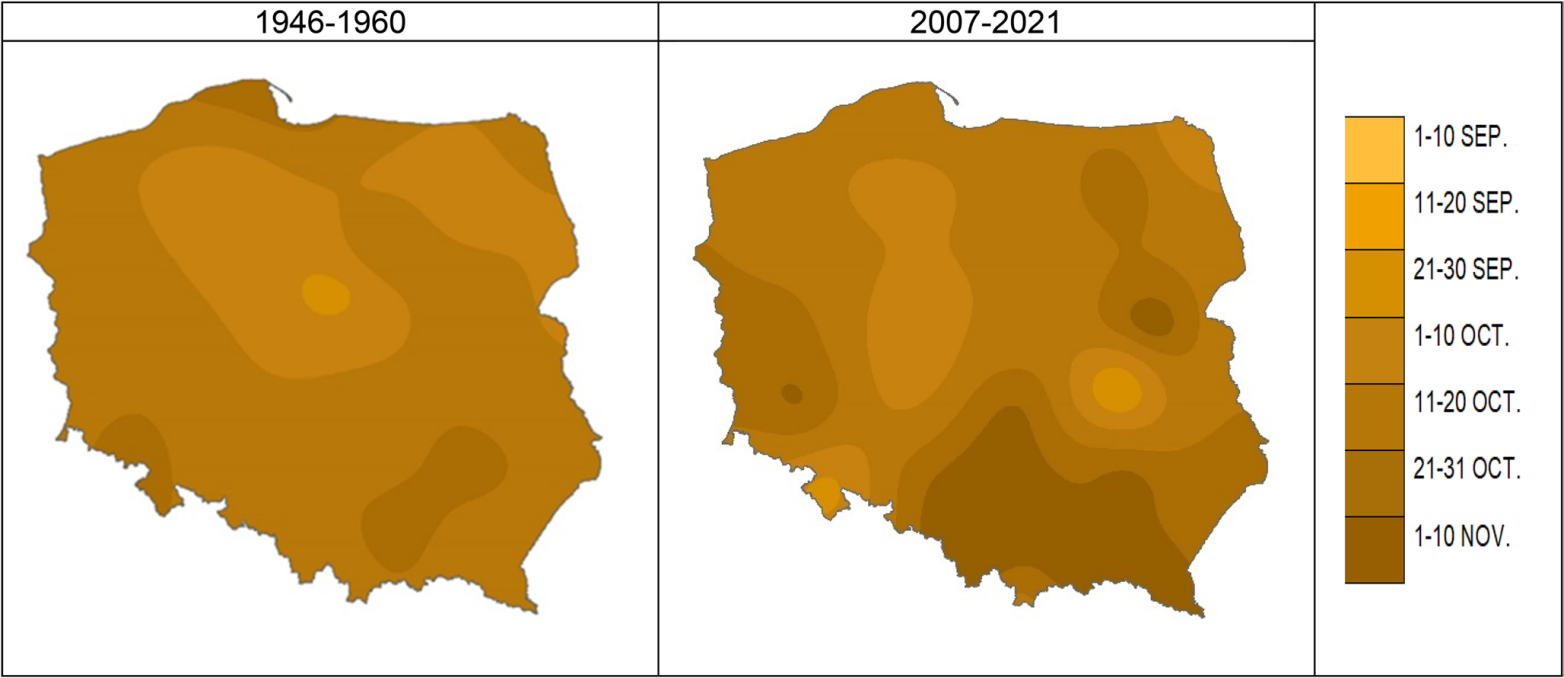
The beginning of the proper autumn in 1946–1960 vs. 2007-2021as [date]; Source: own study

Autumn begins the earliest in the east (northeast), and the latest in the south of Poland (Fig. 3). However, the progression of autumn is less distinct than that of very early spring. Similarly, the variability in the onset of proper autumn in individual years is not as pronounced as in very early spring.

Welch’s t-test was applied to assess whether the observed differences in the mean end dates of the growing season were statistically significant at the 0.05 level, indicated significance in 7 out of the 26 analyzed locations. In five of these, a delay in the end of the growing season was observed, while in two, it occurred earlier (i.e. the arrival of autumn was accelerated). The maximum acceleration of the growing season’s end was recorded in Kłodzko, while its greatest delay in Mikołajki. In both cases, the change amounted to 17 days. Other stations showing a statistically significant (*p* < 0.05) extension of the autumn growing season are located in the eastern part of Poland. In the remaining areas, changes in the end date of the growing season were not statistically significant.

### Growing season duration

**Fig. 4:**
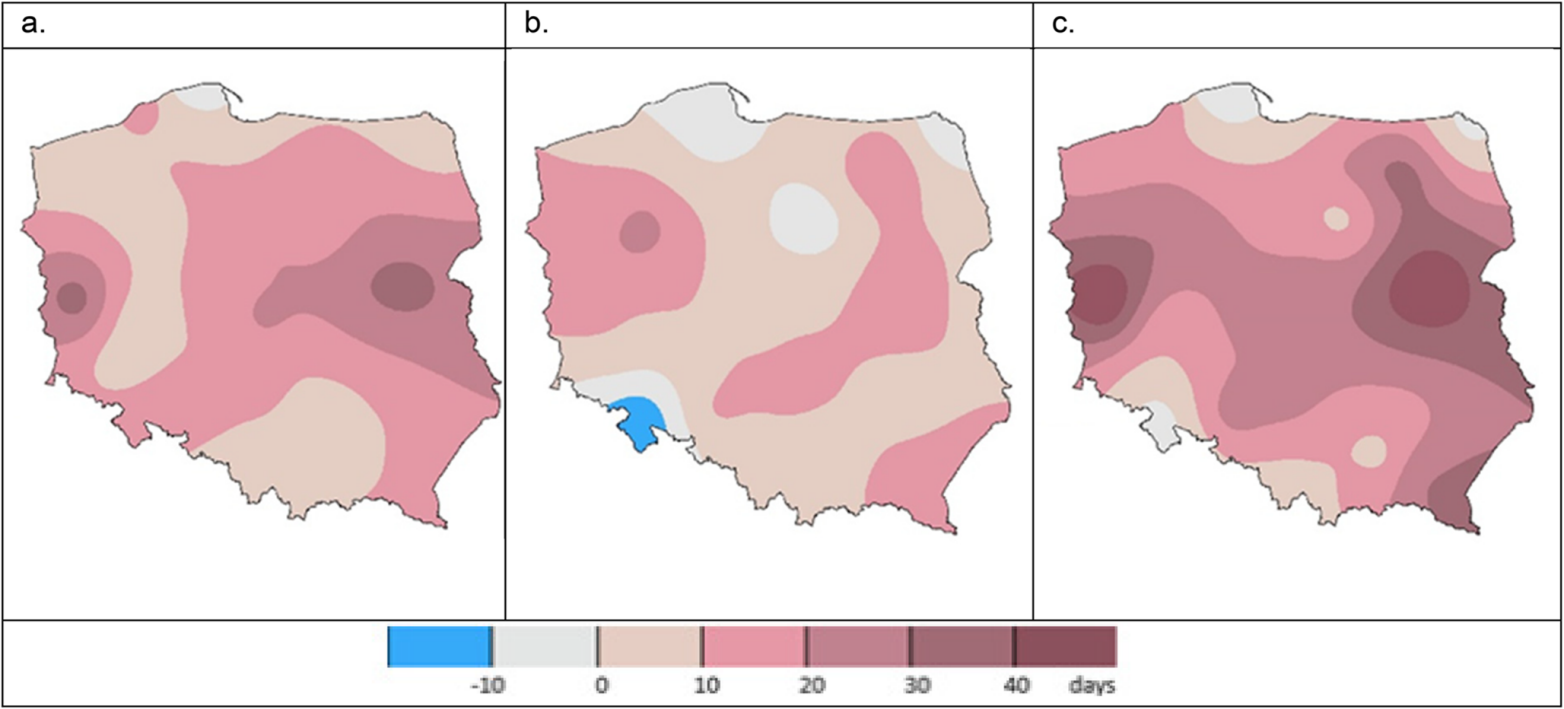
The changes in the growing season duration between the periods 1945–1960 and 2007–2021, driven by: the shifts in the spring vegetation onset (**a**), shifts in the end date of vegetation in autumn (**b**), and the overall change in the growing season length (**c**) in [days]; Source: own study

Changes in the duration of the growing season result from shifts in its start and end dates, with the former having a significantly greater impact – an additional 11 days at the beginning of the growing season, compared to 4 extra days at the end (see Beginning of vegetation and End of vegetation sections). However, a regional approach is essential for accurately identifying these (see Fig. 4).

This study shows that the vegetation period has been shortened by less than 10 days in the Kłodzko Valley and adjacent areas (southwestern Poland), primarily due to an earlier end of vegetation. In the northernmost parts of Poland, the length of the vegetation period is the most stable, with a slight shortening (several days) observed along the western coast. The most significant changes - a substantial extension of the vegetation period (more than 1 month) - were recorded in the central lowlands, mainly driven by an earlier onset of spring. In the highlands (south and southeastern Poland) an extension of the growing season (although to a lesser extent) was also recorded (Fig. 4).

However, in each specific location, the range between the extreme dates of the onset of very early spring in subsequent years can vary (in each of the 15-year periods) from about a month to as much as 50 days. In the record holder Kołbaskowo, the onset of very early spring occurred between the first decade of February and the second decade of April. The range between the extreme dates of the autumn onset in individual years amounts to about a month in specific locations, with the largest — 48 days (from the third decade of September to the first decade of November) — recorded in Nowy Sącz after 2007.

**Fig. 5:**
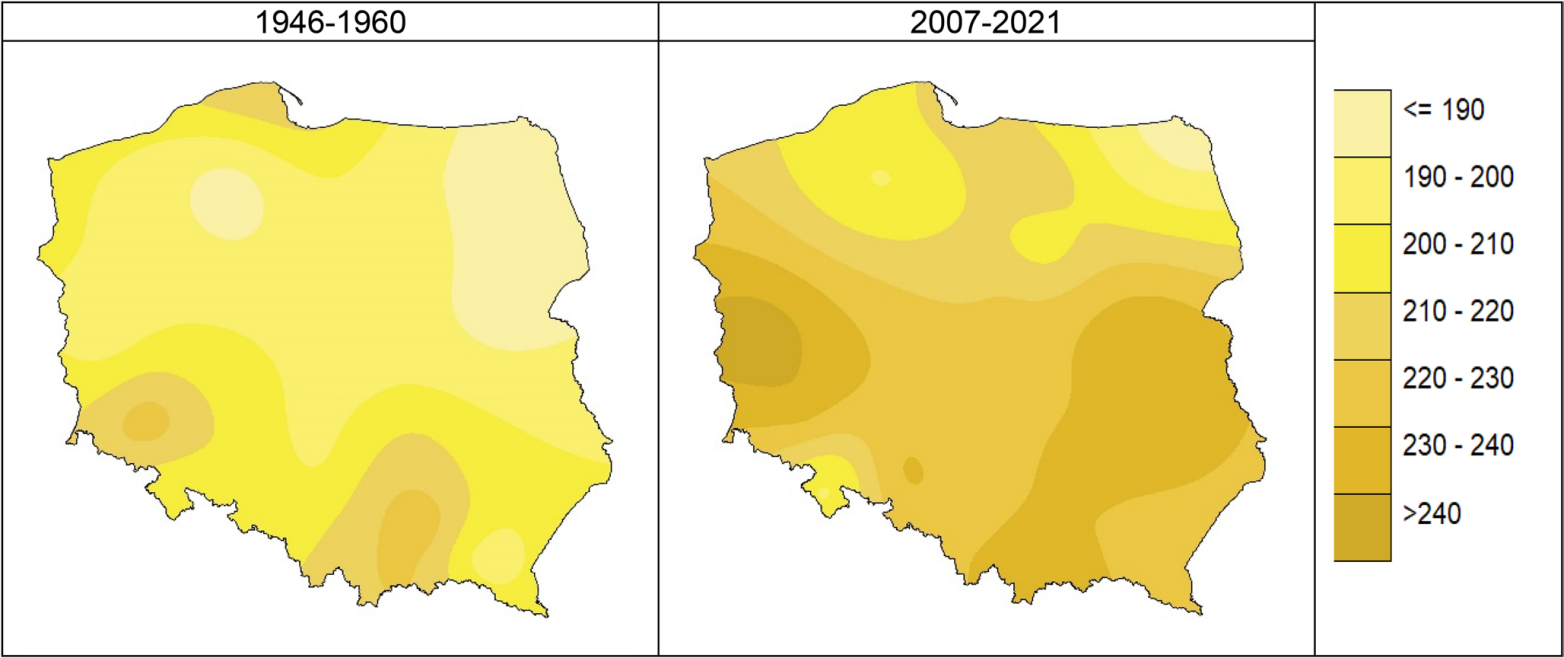
The growing season duration in 1946–1960 vs. 2007–2021 in [days]; Source: own study

The growing season in Poland before 1960 lasted from 185 days in the northeast (generally about 190 days in the east) to 220 or more days in the southwest. As a result of the currently observed climate changes, its duration has increased after 2007 to more than 240 days in the west. In the central lowlands and the Lublin Upland, it has risen to 220–230 days. On the coast and in the northeast of Poland, it stays almost the same, i.e. 185–200 days (Fig. 5).

## Discussion

In the last decades of the 20th century, changes in climatic conditions began to be recorded, both globally and regionally. These changes concern various meteorological elements, but in the temperate climate zone, the most significant are the increased air temperature and the frequency of extreme phenomena. In 2011–2020, the Earth’s average temperature was 1.09 degrees Celsius higher than that in 1850–1900, considered equal to the temperature in pre-industrial times. Each of the last four decades was warmer than its predecessors (IPCC 2021). The average temperature in Poland is also steadily rising (Ustrnul et al. 2021). Poland’s previously “typical season” characteristics are changing along with the tendency towards global warming. The seasons are generally becoming warmer. However, average temperatures rise over time at different rates and significance levels (Szwed and Wasielewska 2024).

Climate change cannot go unnoticed in its impact on other non-climatic elements of the natural environment (including plants). That’s why phenological observations have become an effective tool for monitoring current climate changes, both in terms of their pace and the areas where the effects of these changes are most pronounced. Time shifts in successive stages of plant development (as well as in the onset of subsequent phenological seasons) can clearly demonstrate ongoing climate changes. The first indicator of these changes is the alteration in the duration and cut-off dates of the growing season.

This research revealed an apparent acceleration in the onset of vegetation—by an average of 11 days for Poland (an average value). Before 1960, vegetation started at the end of the first decade of March and covered the country until the second half of April. Currently, it begins as early as the end of February and reaches most of the territory by the end of March. The long-term variability related to the transitional nature of Poland’s climate can explain a significant part of the regional variability. Simultaneously, the results pointed out that only in eight locations, the acceleration of the vegetation is statistically significant at the 0.05 level. The majority of the detected changes are weak. These are tendencies rather than trends. However, omitting information about such a signal resulting from data seems to be carefree. Botanical studies indicate that higher air temperatures in January through March contribute to the earlier flowering of hazel (Siłuch et al. 2016). Conversely, delays in hazel flowering are influenced by thicker snow cover in the early months of the year and lower air temperatures. Climatic studies corroborate these observations. The progressive warming is most noticeable in the cold season, manifested by the shortening of the thermal winter period (Czarnecka and Niezgórska-Lencewicz 2017; Czernecki and Miętus 2015). Kożuchowski and Degirmendžić (2005) note that winters have become milder and less snowy, and springs are warmer than autumns. Along with the reduction in snow cover thickness, the mean number of days with snow cover in winter also decreases over time (e.g., Wibig and Jędruszkiewicz 2023; Tomczyk et al. 2021). Similarly, Kożuchowski and Żmudzka (2001) highlighted seasonal changes, such as the earlier onset of subsequent thermal seasons and marked shortening of winter.

This research shows that the end of the vegetation period is more stable. Although a delay was recorded at 16 out of 26 phenological stations, it averaged only 4 days. The delays are statistically significant only in the case of 5 stations. There are also stations where no changes were observed, or even a slight acceleration of the end of the growing season was recorded (with two stations accelerating more and significantly). These stations are primarily located along the coast and in the eastern part of the country. The progression of proper autumn (marking the end of vegetation) is less distinct compared to the onset of vegetation. The stability of autumn temperatures and lower variability during this season are also highlighted in numerous scientific publications, e.g., Szwed and Wasielewska 2024; Chojnacka-Ożga and Ożga 2018. At the same time, Sparks and Menzel (2002) emphasize that at the end of the growing season, plants are far less responsive to changes in average temperatures. The course of preparation for winter dormancy is influenced by both the conditions prevailing throughout the growing season (e.g., long-term drought accelerating leaf yellowing) and meteorological events of a local nature, such as frosts, strong winds, etc.

An increasingly earlier start of the vegetation period and a slight delay in its end result in an extended growing season. Before 1960, it lasted from 185 days in the northeast to 220 or more days in the southwest. Nowadays, its duration has increased to more than 240 days in the west, while in the central lowlands and the Lublin Upland to 220–230 days. It stays almost the same on the coast and in the northeast of Poland.

The presented results confirmed the thesis about extending the growing season and are consistent with the findings reported in the literature. The message from all phenological and meteorological studies, whether Polish or from other regions, is consistent: the growing season is starting earlier and becoming longer. In Poland, such conclusions are presented by Tomaszewska and Rutkowski (1999), Nieróbca et al. (2013), Chojnacka-Ożga and Ożga (2018), Szyga-Pluta et al. (2023), and Szwed and Holka (2024), etc. On a European scale, similar findings have been reported based on phenological, satellite, and climatological studies, e.g., by Menzel and Fabian (1999), Ahas et al. (2000), Chmielewski and Rőtzer (2002), Stockli and Vidale (2004), Chmielewki et al. (2005), Linderholm et al. (2008), Aalto et al. (2022), Kollo et al. (2023), and among others.

However, comparing the obtained results with specific numerical values describing the duration of the growing season or specific vegetation cut-off dates found in scientific literature is challenging or even impossible due to the differences in methodologies. For instance, various indicator plants are used in phenological studies. At the same time, different methods are employed to determine vegetation cut-off dates in meteorological studies, even when the same temperature threshold for plant development is assumed.

It should be noted, however, that the above general statements pertain to average conditions. Due to its location in the transitional temperate climate zone, Poland is characterized by high temporal variability, i.e. weather variability from day to day, season to season, or between years. The transitional nature of Poland’s climate is also evident in the climatic differences between regions (spatial variability). The overall climatic conditions of a given area are influenced by both regional factors (e.g. altitude above sea level, distance from the sea, etc.) and local factors (e.g. topography, presence of water reservoirs, land use/cover, etc.). However, atmospheric circulation remains the dominant climatic factor. Various air masses converge over Poland, and their influence on regional climates varies. Nevertheless, westerly circulation is particularly important in shaping Poland’s climate. Most often (on approximately 65% of days), moist polar-maritime air masses from the Atlantic reach the country. Over the multi-year period from 1951 to the present, in addition to its considerable fluctuations, a weak but noticeable trend of increasing intensity has been observed (Niedźwiedź and Ustrnul 2021).

Thus, the most visible changes in the duration of the growing season occur in the central lowlands of Poland. These are areas where, in line with the dominant atmospheric circulation, the climate is shaped primarily by ocean (maritime) air masses. Their west-to-east flow is unimpeded by any natural barriers such as mountain ranges, and their influence is steadily increasing. These maritime air masses contribute to the continued mildening of winters and the earlier onset of thermal spring, which in turn leads to an earlier start of the growing season. However, it should be noted that as these air masses penetrate further inland, they gradually lose their maritime characteristics. As a result, their influence is considerably weaker in eastern Poland. The increase in average spring temperatures is the highest among all seasons, as confirmed by studies by Kożuchowski 2011; Ustrnul et al. 2021; and Szwed and Wasielewska (2024). The most pronounced warming is observed in western Poland.

The observed extension of the growing season in the eastern part of the central lowlands is quite surprising. Given that this area remains under the influence of polar-continental climate patterns from the east, where very cold and, more importantly, prolonged winters can occur, such a substantial increase in favorable conditions for vegetation raises some concerns. However, Radzka (2014, 2021) appears to confirm these findings, noting significant temperature increases in this region both at the beginning of the growing season and in the final months (particularly October). This area is also where significant increases in winter temperatures, a notable decline in frost resistance (Szwed and Wasielewska 2024), and a large extension of the frostless period (Ustrnul et al. 2021) have been recorded.

On the other hand, this study found considerable stability in the duration of the growing season in the north. This appears justified, given that the Baltic Sea’s moderating effect plays a crucial role in shaping the climate in this region (especially the weather conditions in winter). As a result, winters along the coast have historically been milder than in other areas, particularly those in the east, which experience strong continental influences. Furthermore, the Baltic Sea also mitigates the effects of ongoing climate change. However, other studies based solely on average daily temperatures indicate a slight increase in the length of the growing season in this area (cf. Szwed and Holka 2024).

Climate changes occurring in the southern part of Poland, particularly in the foothill and mountainous areas, are the most difficult to interpret. In these regions, local conditions influence the climate much more than in other parts of the country. Factors such as topography, altitude, and exposure can significantly differentiate climatic conditions, and consequently, phenological responses, even between closely situated areas. Therefore, as noted in the methodology section, extrapolated values for these regions should be treated with great caution or even considered potentially unreliable. Moreover, as many researchers have pointed out (e.g. Beniston 2003; Pepin et al. 2015; etc.), mountain regions (not only high-altitude zones) are more sensitive to climate change. For this reason, climate change and related environmental transformations (including phenological changes) should be the subject of dedicated research.

##  Conclusions

This study confirms that climate change has a measurable impact on the growing season in Poland. More specifically, it has resulted in an asymmetric season extension, driven primarily by an earlier onset and, to a lesser extent, a later end of the growing season. In some regions, these changes are more pronounced than in others. Regional variability underscores the complexity of phenological responses to changing climatic conditions, which may be shaped by regional and local factors.

The extension of the growing season is undoubtedly positive for plant development/growth, and agricultural production, especially. The obtained results are encouraging from an economic perspective, as the most significant extension of the growing season affects predominantly agricultural areas. A longer growing season brings many benefits for plant production. An earlier sowing date for spring crops maximizes the use of water resources accumulated in the soil during winter. A longer growing season favours the cultivation of thermophilic species such as corn, sunflower, soybean, millet, and sorghum (Marcinkowski and Piniewski 2018). Prolonging the autumn vegetation period is beneficial for sowing winter crops. Favourable temperatures for an extended duration enable timely autumn plant protection treatments against weeds and fungal diseases. Air temperature also significantly impacts the effectiveness of many active substances contained in plant protection products (Czarnecka et al. 2010). Unfortunately, extending the growing season can sometimes have adverse effects, including increased threats to crop plants from weeds, pathogens, and pests (Skendžić et al. 2021).

Finally, it should be noted that although the vast majority of studies, including this one, indicate a progressive extension of the growing season in Europe due to global warming, some opinions suggest that this trend may not continue. Rahmati et al. (2023) argue that the increasing trend has reversed over the past decade, with the length of the growing season returning to 1980 s levels due to increased transpiration demands. All the more so, it is essential to continue phenological observations of selected plants to obtain longer observation series. We do not know the full consequences of climate change. Thus, it is essential to remain vigilant and consistently monitor the upcoming changes, particularly in the context of further projected climate shifts.

The authors are aware of the many shortcomings of the presented study. Relatively short data series, the selection of observation stations, and particularly the matching of current stations to historical ones, may be subject to criticism. The short 15-year phenological data series limit the use of statistical methods for trend analysis and, consequently, the ability to draw conclusions about the significance of observed changes in the timing of individual phenophases and the growing season cut-off dates. Nevertheless, considering the importance of the issue, even if the presented results are treated as illustrative or preliminary, they provide valuable insights into the study on “recognizing climate change” and allow for preliminary conclusions about the observed changes.

## Data Availability

The phenological data that support the findings of this study stem from the database of the Institute of Meteorology and Water Management–National Research Institute (IMGW-PIB), and they are not available. The meteorological data are openly available in the IMGW-PIB Public Data Repository (https://dane.imgw.pl/).
